# Effect of high-salt diet on blood pressure and body fluid composition in patients with type 1 diabetes: randomized controlled intervention trial

**DOI:** 10.1136/bmjdrc-2019-001039

**Published:** 2020-05-12

**Authors:** Eliane F E Wenstedt, Nienke M G Rorije, Rik H G Olde Engberink, Kim M van der Molen, Youssef Chahid, A H Jan Danser, Bert-Jan H van den Born, Liffert Vogt

**Affiliations:** 1 Department of Internal Medicine, Amsterdam UMC - Locatie AMC, Amsterdam, North Holland, Netherlands; 2 Department of Pharmacy, Amsterdam UMC - Locatie AMC, Amsterdam, North Holland, Netherlands; 3 Department of Internal Medicine, Erasmus MC, Rotterdam, Zuid-Holland, Netherlands

**Keywords:** extracellular fluid volume, hypertension, plasma volume, salt, salt sensitivity, sodium, systemic vascular resistance, type 1 diabetes, vasodilation

## Abstract

**Introduction:**

Patients with type 1 diabetes are susceptible to hypertension, possibly resulting from increased salt sensitivity and accompanied changes in body fluid composition. We examined the effect of a high-salt diet (HSD) in type 1 diabetes on hemodynamics, including blood pressure (BP) and body fluid composition.

**Research design and methods:**

We studied eight male patients with type 1 diabetes and 12 matched healthy controls with normal BP, body mass index, and renal function. All subjects adhered to a low-salt diet and HSD for eight days in randomized order. On day 8 of each diet, extracellular fluid volume (ECFV) and plasma volume were calculated with the use of iohexol and ^125^I-albumin distribution. Hemodynamic measurements included BP, cardiac output (CO), and systemic vascular resistance.

**Results:**

After HSD, patients with type 1 diabetes showed a BP increase (mean arterial pressure: 85 (5) mm Hg vs 80 (3) mm Hg; p<0.05), while BP in controls did not rise (78 (5) mm Hg vs 78 (5) mm Hg). Plasma volume increased after HSD in patients with type 1 diabetes (p<0.05) and not in controls (p=0.23). There was no significant difference in ECFV between diets, while HSD significantly increased CO, heart rate (HR) and N-terminal pro-B-type natriuretic peptide (NT-proBNP) in type 1 diabetes but not in controls. There were no significant differences in systemic vascular resistance, although there was a trend towards an HSD-induced decrease in controls (p=0.09).

**Conclusions:**

In the present study, patients with type 1 diabetes show a salt-sensitive BP rise to HSD, which is accompanied by significant increases in plasma volume, CO, HR, and NT-proBNP. Underlying mechanisms for these responses need further research in order to unravel the increased susceptibility to hypertension and cardiovascular disease in diabetes.

**Trial registration numbers:**

NTR4095 and NTR4788.

**Significance of this study**

**What is already known about this subject?**

- Patients with type 1 diabetes are more susceptible to hypertension and increased cardiovascular risk.
- Increased salt sensitivity may underlie this phenomenon; however, evidence is scarce and conflicting.

**What are the new findings?**

- Young, normoalbuminuric and normotensive patients with type 1 diabetes are more salt-sensitive compared with matched healthy individuals.
- The salt-sensitive blood pressure (BP) increase in patients with type 1 diabetes was accompanied by significant increases in plasma volume, cardiac output, heart rate, and N-terminal pro-B-type natriuretic peptide.

**How might these results change the focus of research or clinical practice?**

- These findings underline the benefit of dietary salt restriction in patients with type 1 diabetes to control BP.
- Future research is required to assess potential mechanisms underlying the observed responses to salt in patients with type 1 diabetes.

## Introduction

Type 1 diabetes is associated with an increased risk of hypertension and hypertension-mediated complications.^1 2^ In a large cohort study, hypertension prevalence in patients with type 1 diabetes was estimated to be 43% compared with 15% in healthy controls matched for age and sex.^3^ The increased susceptibility to hypertension may result from increased sensitivity to high salt (NaCl) intake. Although hypertensive patients with type 1 diabetes do not appear to be more salt-sensitive than patients with hypertension in general,^4^ there is evidence that *normotensive* patients with type 1 diabetes are more salt-sensitive compared with control subjects matched for age, gender, and body mass index (43% vs 17% that were classified as salt-sensitive, which was defined as a ≥3 mm Hg increase in mean arterial pressure (MAP)).^5^ However, there are also studies that suggest that salt sensitivity is more or only apparent in case of microalbuminuria.^6–8^

Increased sodium retention by the kidney is thought to be the principal determinant of the relation between high-salt intake and hypertension, by leading to an increase in extracellular fluid volume (ECFV) and stroke volume, resulting in a subsequent rise in blood pressure (BP).^9^ Patients with type 1 diabetes are suggested to have alterations in body fluid composition (including increased venous blood volume^10^ and increased ECFV^11 12^) and might respond differently to high-salt consumption in terms of ECFV or plasma volume compared with healthy individuals. However, studies that support this assumption have never been conducted. Moreover, importantly, there is an increasing body of literature that questions the validity of the classical volume theory.^13–16^

Therefore, a careful assessment of responses in patients with type 1 diabetes to dietary high salt is warranted.

The primary aim of the present study was to investigate the effect of salt on BP and body fluid composition (ie, ECFV and plasma volume) in patients with type 1 diabetes. Additionally, the effect of salt on cardiac output (CO) and systemic vascular resistance was assessed.

## Research design and methods

### Participants

We carried out two identical randomized cross-over intervention studies in patients with type 1 diabetes and healthy controls, respectively. Male, non-smoking individuals between 18 and 40 years old who were able to provide written informed consent were included. Patients with type 1 diabetes had to have a normal and stable renal function and stable hemoglobin A1c levels between 6% and 10% (42–86 mmol/mol) during the six months preceding the study. Use of renin–angiotensin system blocking agents was allowed for patients with type 1 diabetes, but these were discontinued prior to the study visits for five times the elimination half-life. Insulin doses were kept stable throughout the whole study period. We excluded overweight subjects (body mass index>30 kg/m^2^), subjects with a BP of 140/90 mm Hg or higher, and subjects with decreased kidney function (estimated glomerular filtration rate<60 mL/min). The trials were conducted in accordance to the original protocols (www.trialregister.nl) and the reporting adheres to the Consolidated Standards of Reporting Trials guidelines.^17^

### Study design and measurements

The primary endpoint of this study was ECFV. Secondary endpoints were plasma volume and hemodynamics (ie, systemic vascular resistance and CO, consisting of stroke volume and heart rate (HR)). Our null hypothesis was that there would be no difference in BP and body fluid composition between diets. Salt loading was pursued by means of a dietary protocol, which is considered to be the current reference method for testing the effects of salt.^18^ All subjects adhered to an 8-day low-salt diet (LSD) (<3 g NaCl/day) and high-salt diet (HSD) (>12 g NaCl/day) in randomized order and a time period of 1–2 weeks with a normal diet in-between. Diet order was determined by block randomization via sealed envelopes by the study investigators, and diets were not masked for the study subjects or investigators during follow-up. Diets were pursued with the help of a dietary list, which advised to resemble the normal diet of the individual as much as possible, for example, by adding extra salt instead of changing the whole dietary pattern. We checked dietary compliance by collecting 24-hour urine samples on day 3, 6 and 8. The measurements on day 8 were used for analysis and are depicted in the tables. Also, on day 8 (the last day of the diet), blood sampling and hemodynamic measurements were performed. Plasma–renin activity (PRA) was measured by enzyme-kinetic assay as described before.^19^ Aldosterone was measured by radioimmunoassay (Demeditec Diagnostics, Kiel, Germany).^20^ Brachial BP was measured in supine position with a semiautomatic device (Omron 705 IT, OMRON Healthcare, The Netherlands) after at least 10 min of supine rest in a quiet and temperature-controlled room. We performed five sequential measurements and used the mean of last two readings for analysis. MAP was calculated by the sum of two-thirds∙(diastolic BP) and one-third∙(systolic BP). CO and HR were measured after at least 15 min of supine rest with the Nexfin device (Edward Lifesciences BMEYE B.V., Amsterdam, the Netherlands). This device determines stroke volume using the pulse contour method (Nexfin CO-trek) and divides it by the interbeat interval to calculate CO.^21^ The parameters are determined from the average of a 30 s stable recording period. Systemic vascular resistance (SVR) was calculated by dividing the MAP by CO. Solute-mediated water clearance and solute-free water clearance were calculated from the urinary osmolality, total urine volume, and plasma osmolality, and electrolyte-free clearance was calculated from urinary sodium and potassium concentrations, total urine volume, and plasma osmolality.^22^ These clearances were used to assess to what extent the diuresis is driven by urinary solutes or electrolytes. Subjects were instructed to refrain from alcohol intake and heavy physical exercise 24 hours prior to the study visit and to avoid caffeine intake 12 hours in advance.

### ECFV and plasma volume

At the study visit, two intravenous catheters were placed in the left and right antecubital veins. Iohexol (Omnipaque 647 mg iohexol/mL), a non-ionic radiopaque contrast agent that is distributed throughout the whole extracellular space, was used to measure ECFV according to the method by Zdolsek *et al*.^23^ We administered 10 mL iohexol at day 8 of both diets through a venous catheter. Because of relatively rapid elimination by the kidneys, a reasonably steady state is never reached, and continuous elimination of iohexol must be considered in the calculations. Therefore, blood was drawn at regular time intervals after infusion of iohexol (t=0, 5, 10, 15, 30, 60, 90, 120, 150, 180 and 240 min after infusion). For ECFV calculation, a two-compartment kinetic model with an expected distribution phase of approximately 20 min was fitted to the data.^23^ The obtained values were multiplied by 0.934 to account for the water content of plasma.^24^

Plasma volume was measured by labeled human serum albumin (^125^I-albumin). A ^125^I-albumin solution of 100 kBq in 5 mL saline was administered intravenously. Blood samples were drawn at the contralateral arm at regular time intervals after infusion (t=0, 5, 10, 15, 20, 30, 45 and 60 min after infusion). One urine sample was obtained at t=60 min. Plasma radioactivity was measured in the blood and urine samples using a scintillation detector (Wizard^2^ 2480 Automatic Gamma Counter (PerkinElmer, USA), measuring in duplicate with a coefficient of variation of <3%. The routine quality control tasks of the gamma counter were performed according the standard Good Laboratory Practice (GLP) features of PerkinElmer, including detector energy resolution, background, absolute and relative detector efficiency, detector stability probability and calibration. Plasma volume was determined by calculating the y-intercept of the disappearance curve of ^125^I-albumin, corrected for the injected dose of tracer, according to the method described by van Kreel *et al.*^25^

ECFV and plasma volume calculations were done with PKSolver, a free Microsoft Excel add-in validated for pharmacokinetic (PK) and pharmacodynamic data analysis.^26^

### Statistical analysis

Data were expressed as mean with SE or SD for parametric and median with IQR for non-parametric variables. Differences between LSD and HSD were assessed using paired t-tests for parametric distributions and Wilcoxon rank-sum test for non-parametric distributions. To assess differences between patients with type 1 diabetes and healthy controls, unpaired t-tests and Mann-Whitney tests were used. The presence of time order and carry-over effects was tested by comparing the means between the two diet orders.^27^ To test for associations, Pearson’s or Spearman’s correlation coefficient for parametric or non-parametric data were used. All statistical analyses were performed using IBM SPSS Statistics V.22.0. A p value of <0.05 was considered significant.

### Sample size calculation

We used anticipated changes in BP and body weight for the sample size calculation, with the latter serving as a proxy for ECFV, since there were no data on measured ECFV after HSD.

As for the patients with type 1 diabetes, we calculated that a sample size of five subjects would have 80% power to detect a difference in BP of 4 mm Hg between a LSD and HSD (based on a two-sided t-test, alpha error of 5%) using data from the study of Strojek *et al*.^5^ In a pilot experiment, we showed that, after changing from an LSD to an HSD, the difference in body weight was 1.7 (SD 1.0) kg between subjects on an LSD and an HSD, according to which we calculated that at least six subjects would be needed for each group (based on a two-sided t-test; power of 80%, alpha error of 5%). Taking into account a possible drop-out of subjects after inclusion, we decided to include at least eight patients with type 1 diabetes.

As for the healthy subjects, at least six subjects were needed for each group to demonstrate a 1.7 (SD 1.0) kg body weight difference between subjects on LSD and HSD (based on a two-sided t test; power of 80%, alpha error of 5%). We demonstrated a 5 mm Hg (SD 5) systolic BP difference between healthy subjects in our pilot on LSD and HSD, indicating that at least 10 subjects were needed (two-sided t-test; power of 80%, alpha error of 5%). Taking into account possible drop-out of subjects after inclusion, we decided to include at least 12 healthy subjects. Of this trial, one article was published previously.^28^

## Results

### Population and dietary intervention

We screened nine patients with type 1 diabetes (between March 2015 and November 2015) and 19 healthy controls (between March 2013 and Augustus 2014). Of the patients with type 1 diabetes, one patient had to be excluded due to a body mass index of >30 kg/m^2^. Of the healthy controls, four subjects withdrew their consent after inclusion before randomization and three subjects were excluded before randomization (one due to high BP and two others due to difficulties with blood drawing). Therefore, we included eight patients with type 1 diabetes and 12 healthy controls with mean ages of 28 (SD 6) and 22 (SD 4) years, respectively, with a normal and similar BP, body mass index, and renal function. Detailed baseline characteristics (determined at a screening visit before commencement of the diets) are depicted in online supplementary table 1. There was no loss to follow-up, and all subjects were included in our analyses. All subjects adequately followed dietary instructions, as assessed by 24-hour urine sodium excretion. Mean (SD) 24-hours urine sodium excretion of patients with type 1 diabetes and healthy controls was 23 (SD 13) mmol and 19 (SD 10) mmol after LSD, and was 353 (SD 73) mmol and 341 (SD 104) mmol after HSD, respectively (table 1). One of the eight patients with type 1 diabetes used a renin–angiotensin system blocking agent (lisinopril), which was temporarily discontinued during the study (as indicated in the Research design and methods section). No other medication (except insulin for the patients with type 1 diabetes) was used by the study subjects.

10.1136/bmjdrc-2019-001039.supp1 Supplementary data

**Table 1:** Data are depicted as mean (SD), unless marked with * (median and 95% CI of the median)

	Patients with type 1 diabetes (n=8)			Healthy controls (n=12)		
	LSD	HSD	P value	LSD	HSD	P value
Weight (kg)	75.6 (8.8)	78.2 (9.6)	<0.001	74.0 (6.6)	76.5 (6.7)	<0.001
Extracellular fluid volume (L)	14.6 (2.2)	16.1 (2.8)	0.32	15.1 (3.6)	17.1 (2.6)	0.09
Plasma volume (L)	3.2 (0.5)	3.5 (0.6)	<0.05	3.4 (0.6)	3.6 (0.6)	0.23
Hemodynamics						
Systolic BP (mm Hg)	120.0 (4.5)	126.4 (7.4)	<0.05	117.3 (7.8)	118.8 (5.5)	0.33
Diastolic BP (mm Hg)	60.4 (5.2)	63.6 (5.0)	<0.05	58.3 (5.4)	57.4 (5.4)	0.28
MAP (mm Hg)	80.3 (3.5)	84.6 (5.1)	<0.05	78.0 (4.8)	77.8 (4.8)	0.85
Cardiac output (L/min)	6.6 (0.8)	7.2 (0.7)	<0.05	6.5 (1.0)	7.0 (1.3)	0.10
Heart rate (beats/min)	57.5 (10.1)	63.1 (10.7)	<0.01	54.7 (8.0)	58.9 (11.2)	0.06
Stroke volume (mL)	116.2 (14.3)	116.7 (18.9)	0.87	118.9 (12.0)	120.5 (8.4)	0.67
Systemic vascular resistance (dyn·s·cm^−5^)	991.3 (127.6)	940.2 (94.6)	0.21	983.4 (174.0)	907.3 (154.6)	0.09
Plasma						
PRA (pmol AngI/mL/hour)*	0.48 (0.24–0.70)	0.08 (0.04–0.13)	<0.01	0.36 (0.17–0.63)	0.04 (0.00–0.07)	<0.001
Aldosterone (pg/mL)*	142.6 (130.7–272.9)	34.4 (20.8–52.1)	<0.001	204.2 (49.3–455.3)	28.0 (15.2–64.2)	<0.001
Aldosterone:PRA ratio* (pg/mL/pmol AngI/mL/hour)	699.5 (216.1–959.8)	667.7 (415.8–5067)	0.31	410.4 (361.6–512.9)	443.3 (263.0–566.7)	0.68
NT-proBNP (ng/L)	10.3 (6.2)	51.8 (45.4)	<0.05	12.7 (13.4)	20.8 (15.7)	0.15
Sodium (mmol/L)	137.3 (2.4)	139.8 (2.1)	<0.05	137.5 (1.6)	140.3 (1.8)	<0.01
Potassium (mmol/L)	4.3 (0.3)	4.2 (0.3)	0.41	3.9 (0.3)	3.9 (0.2)	0.76
Chloride (mmol/L)	98.0 (2.3)	101.9 (1.6)	<0.05	99.6 (1.5)	103.3 (2.0)	<0.001
Bicarbonate (mmol/L)	25.2 (2.5)	25.6 (2.0)	0.77	25.5 (2.0)	25.4 (1.6)	0.88
Creatinine (μmol/L)	75.1 (10.1)	70.6 (9.9)	0.12	83.9 (10.0)	77.0 (9.2)	<0.001
eGFR (CKD-EPI)	116.9 (10.6)	120.9 (10.2)	0.08	111.7 (14.1)	119.9 (11.7)	<0.01
Osmolality (mOsm/kg)	290.3 (2.1)	297.6 (6.1)	<0.01	284.9 (3.1)	289.6 (3.8)	<0.01
Glucose (mmol/L)	10.7 (4.0)	11.0 (2.9)	0.88	4.9 (0.3)	5.0 (0.4)	0.70
24-hour urine						
Volume (mL/24 hours)	2246 (1044)	2809 (788)	<0.05	1702 (551)	1909 (544)	0.24
Osmolality (mOsm/kg)	447.1 (209.5)	650.3 (222.1)	<0.05	430.9 (164.4)	743.7 (164.8)	<0.001
Creatinine (mmol/24 hours)	16.5 (2.6)	17.9 (3.4)	<0.05	15.8 (2.4)	17.3 (2.5)	<0.05
Fe_Na_ (%)	0.1 (0.03)	1.0 (0.2)	<0.001	0.1 (0.03)	1.1 (0.5)	<0.001
Sodium (mmol/24 hours)	23.3 (13.0)	352.7 (72.6)	<0.001	19.1 (9.5)	340.8 (104)	<0.001
Potassium (mmol/24 hours)	113.8 (36.3)	105.8 (38.9)	0.51	90.4 (25.4)	89.5 (19.7)	0.93
Solute-free water clearance (L/day)	−0.8 (1.6)	−3.0 (1.8)	<0.01	−0.7 (0.7)	−2.8 (0.5)	<0.001
Solute-mediated water clearance (L/day)	3.1 (0.8)	5.8 (1.8)	<0.001	2.4 (0.5)	4.7 (0.6)	<0.001
Electrolyte-free water clearance (L/day)	1.2 (0.9)	−0.5 (1.0)	<0.001	0.9 (0.6)	−1.2 (0.3)	<0.001

Data are tested using paired t-test (LSD vs HSD) or Wilcoxon test if marked with *.

BP, blood pressure; CKD-EPI, Chronic Kidney Disease Epidemiology Collaboration; eGFR, estimated glomerular filtration rate; Fe_Na_, fractional excretion of sodium; HSD, high-salt diet; LSD, low-salt diet; MAP, mean arterial pressure; NT-proBNP, N-terminal pro-B-type natriuretic peptide; PRA, plasma–renin activity.

### Patients with type 1 diabetes demonstrate a salt-sensitive BP increase

**Figure 1:**
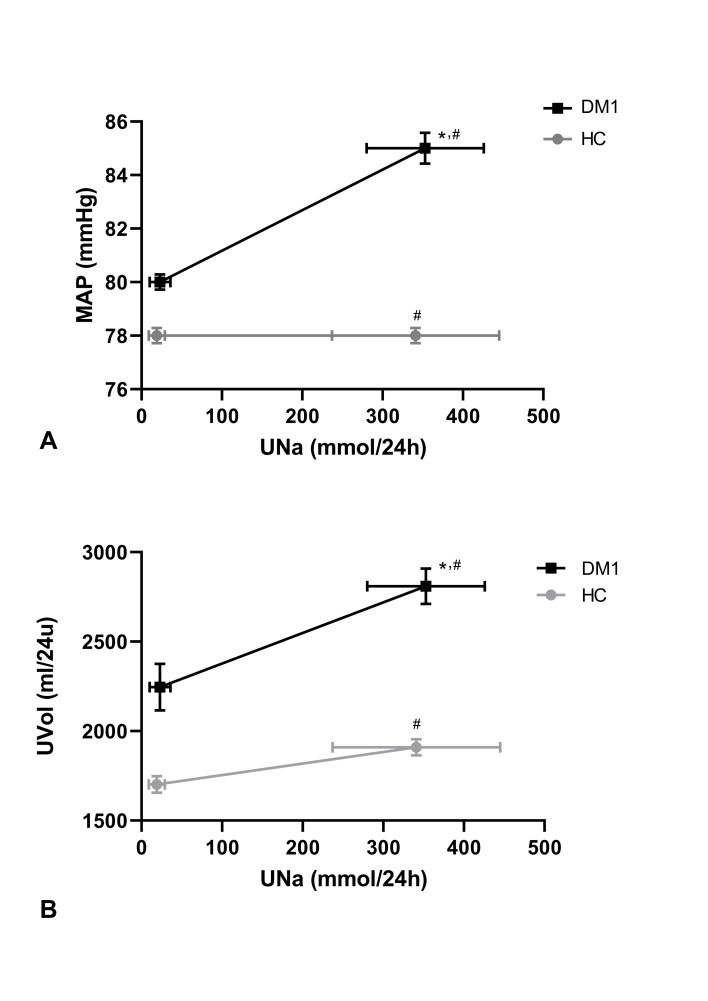
Differences in UNa versus differences in map and UVol between LSD and HSD. (A) Similar increases in UNa in the patients with DM1 and the HCs coincided with distinct MAP responses. (B) Similar increases in UNa in the patients with DM1 and the HCs coincided with distinct UVol responses. Data are presented as mean with SEM and were tested with a paired t-test. *P<0.05 MAP/UVol HSD versus LSD; ^#^p<0.05 UNa HSD vs LSD. DM1, type 1 diabetes; HC, healthy control; HSD, high-salt diet; LSD, low-salt diet; MAP, mean arterial pressure; UNa, urinary sodium; UVol, urinary volume.

In patients with type 1 diabetes, MAP was significantly higher after HSD than after LSD (mean 84.6 (SD 5.1) mm Hg vs 80.3 (SD 3.5) mm Hg, p=0.03; figure 1A and table 1), while MAP was similar after HSD and LSD in healthy controls (77.8 (SD 4.8) mm Hg vs 78.0 (SD 4.8) mm Hg, p=0.85; figure 1A and table 1). Likewise, systolic BP and diastolic BP were higher after HSD in patients with type 1 diabetes but not in healthy controls (table 1). The BP rise in patients with type 1 diabetes did not coincide with a higher difference in 24-hour urinary sodium excretion between LSD and HSD (figure 1A), and BP changes were not correlated to plasma sodium or urinary sodium excretion. Furthermore, the BP rise in patients with type 1 diabetes coincided with increased diuresis after HSD (figure 1B and table 1). Solute-free water clearance and electrolyte-free water clearance decreased after HSD, whereas solute-mediated water clearance increased, all to a similar extent in both groups (table 1).

### Responses to HSD in patients with type 1 diabetes and controls

**Figure 2:**
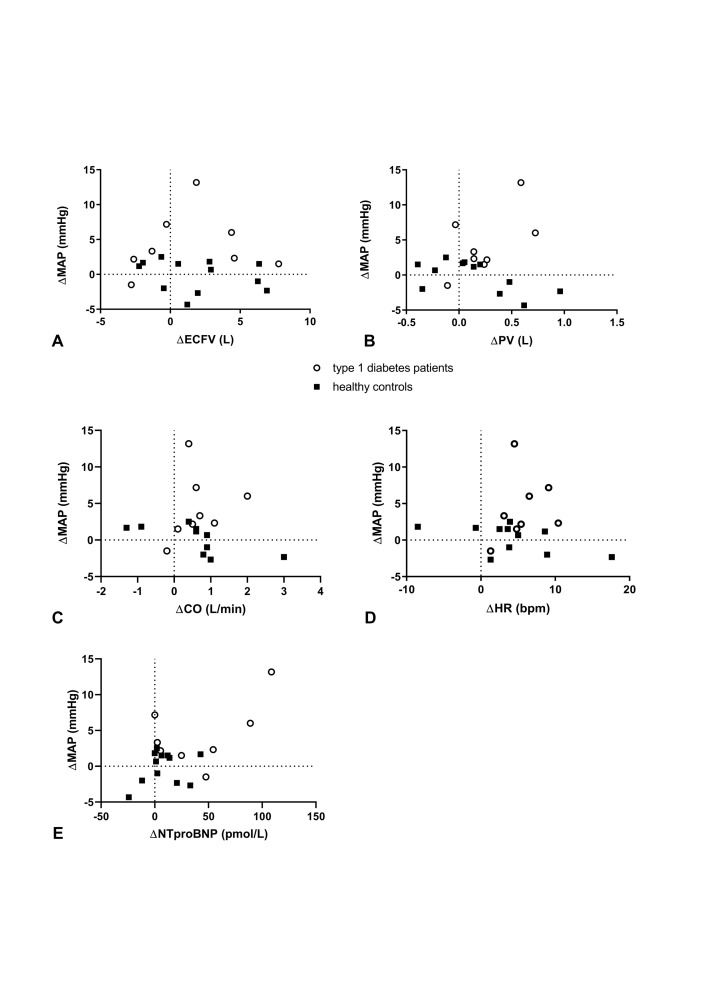
Correlation analyses between MAP and volume-dependent and hemodynamic parameters. (A) There was no correlation between changes (HSD–LSD) in ECFV and MAP (patients with type 1 diabetes: r=0.19, p=0.66; healthy controls: r=−0.31, p=0.33). (B) There was no correlation between changes (HSD–LSD) in plasma volume and MAP (patients with type 1 diabetes: r=0.38, p=0.36; healthy controls: r=−0.54, p=0.08). (C) There was a correlation between changes (HSD–LSD) in CO and MAP in healthy controls (r=−0.92, p<0.01) but not in patients with type 1 diabetes (r=0.47, p=0.24). (D) There was no correlation between changes (HSD–LSD) in HR and MAP (patients with type 1 diabetes: r=0.24, p=0.57; healthy controls: r=−0.51, p=0.11). (E) There was no correlation between changes (HSD–LSD) in NT-proBNP and MAP (patients with type 1 diabetes: r=0.17, p=0.70; healthy controls: r=0.08, p=0.80). CO, cardiac output; ECFV, extracellular fluid volume; HR, heart rate; HSD, high-salt diet; LSD, low-salt diet; MAP, mean arterial pressure; NT-proBNP, N-terminal pro-B-type natriuretic peptide; PV, plasma volume.

In patients with type 1 diabetes, HSD induced significant increases in body weight, BP, CO, HR, N-terminal pro-B-type natriuretic peptide (NT-proBNP), and plasma volume but not in stroke volume or ECFV (table 1). In these patients, there was no correlation between changes in BP and changes in either CO, HR, plasma volume, ECFV, or NT-proBNP (figure 2). In healthy controls, HSD induced significant increases in body weight but not in BP, CO, HR, NT-proBNP, plasma volume, stroke volume or ECFV (table 1). There was an inverse correlation between HSD-induced changes in BP and HSD-induced changes in CO in this group (figure 2). PRA and aldosterone showed a significant decrease after HSD in both groups (p<0.05). HSD did not change the aldosterone/PRA in both groups. Also, changes in PRA and aldosterone were not correlated to changes in BP or plasma volume.

## Conclusions

In the present study, we demonstrate that young, normoalbuminuric and normotensive male patients with type 1 diabetes show a salt-sensitive BP response to HSD, which is absent in matched healthy controls. Although both groups showed a mean increase in plasma volume, CO, NT-proBNP, and HR, responses were more homogeneous and, accordingly, only statistically significant in the patient group with type 1 diabetes, despite the smaller group size. Stroke volume as well as the aldosterone:PRA ratio did not change in response to HSD in both groups.

Despite the mean±2 L HSD-induced ECFV increases in our study, these increases did not reach statistical significance. Although this may be the result of a power problem, the change appeared to be similar in both groups and therefore does not seem to have caused the differential BP response. This is in line with the absence of a correlation between ECFV and BP changes. The ±2.5 kg increase in body weight after HSD in both groups may reflect the ECFV increase, yet contributions of increases in intracellular fluid volume or body fat mass^29^ cannot be excluded with certainty. It may be noted that a recent study did not find any difference in ECFV after 1-week dietary salt loading in healthy individuals, in which the difference between LSD and HSD approximated 8 g (vs ±18 g in our study).^30^ The similarity in ECFV in patients with type 1 diabetes and healthy controls on LSD and HSD is consistent with previous studies in normoalbuminuric patients with type 1 diabetes, since only in the presence of advanced chronic kidney disease ECFV was increased.^11 31^

The mechanisms underlying the observed plasma volume response in patients with type 1 diabetes need further exploration. It may reflect increased sodium retention caused by (exogenously administered) insulin, given its sodium-retaining effects.^32^ The extent of volume retention in response to salt is also interlinked with the extent to which individuals are able to store sodium without concurrent water retention in certain compartments of their body.^33^ Titze *et al* has shown that a significant amount of sodium can be stored in tissues like the skin, in concentrations that far exceed plasma levels.^34^ High interstitial sodium content is known to be present in a variety of salt-sensitive conditions like type 2 diabetes,^35^ chronic kidney disease,^36^ and hypertension,^37^ and may preclude further sodium buffering in response to a salt overload. Whether there is an effect of exogenous insulin on sodium storage is yet unknown but may be worth exploring.^32^ It remains to be determined whether in patients with type 1 diabetes reduced sodium storage capacity in response to salt loading underlies their response in plasma volume. Volume retention was also shown to be associated with vasodysfunction in salt-sensitive healthy individuals.^38^ Laffer *et al* showed that salt-resistant individuals show a decrease in systemic vascular resistance after salt loading by mechanisms not yet completely elucidated but possibly involving nitric oxide-related effects or neural pathways.^38 39^ In the present study, a significant decrease in systemic vascular resistance could, however, not be demonstrated, possibly due to the heterogeneity in BP responses in healthy controls (ie, seven out of 12 healthy controls showing a BP increase and five showing a decrease) (table 1). However, the trend towards a decrease in systemic vascular resistance in healthy controls and the absence of a decrease in patients with type 1 diabetes might point to vasodysfunction in the latter, which would be in accordance with recent concepts, proposing that vasodysfunction rather than impaired renal sodium excretion is the key causal factor in a salt-sensitive BP rise.^14 38^ The nature of the increase in NT-proBNP may lie in pressure overload as well as volume expansion, although we could not demonstrate a correlation between changes in NT-proBNP and BP, systemic vascular resistance, or plasma volume in the present study (data not shown).

The HSD-induced increase in HR in our study is surprising and in contrast with a recent meta-analysis of 63 randomized controlled trials that showed a 2.4% increase in HR with salt *reduction* rather than with salt loading.^40^ Reasons for this remain to be established, but may involve differences in measurement methods or conditions (in our case, non-invasive continuous hemodynamic monitoring after at least 15 min of supine rest), study population, or the nature of the salt intervention. The higher plasma osmolality in type I diabetes may have caused the HR increase, as it has been shown that plasma osmolality rises increase sympathetic nerve activity.^41 42^

Finally, HSD increased 24-hour urine volume in patients with type 1 diabetes but not in healthy controls. As the increased diuresis was not accompanied by differences in changes of solute-free water clearance, solute-mediated water clearance, or electrolyte-free water clearance compared with healthy controls, differences may be caused by differences in fluid intake. In both groups, HSD resulted in an increase in free water retention as evidenced by the negative solute- and electrolyte-free water clearance, likely reflecting osmotically stimulated vasopressin release.

With regard to the excessive cardiovascular risk of patients with type 1 diabetes, which is importantly mediated by hypertension development,^43^ our data bear clinical importance. It underlines the importance of avoidance of excessive salt intake, as recommended in this patient group.^43^ Also, the observed increased sensitivity to salt may contribute to the increased cardiovascular risk of these patients even independent of BP, since it has been shown that individuals who are normotensive on the longer term but have been previously tested as salt sensitive show increased mortality, although by mechanisms still unknown.^44^ It should be noted that the extent of salt reduction needs further research. Paradoxically, a 1-week LSD has been demonstrated to induce relative hyperfiltration in the kidney in patients with type 1 diabetes.^8^ Also, cohort studies showed increased mortality with highest but also with lowest salt intake in patients with type 1 diabetes^45^ and in patiens with type 2 diabetes,^46^ although possibly hampered by inaccurate estimations of salt intake and confounding.^47 48^

The major strength of our study is that—to our knowledge—we are the first to measure body fluid composition in conjunction with BP after salt loading in patients with type 1 diabetes using validated and precise PK methods, instead of relying on estimations based on body weight or bioimpedance methods that can be troubled by several factors.^49^ Furthermore, we included a well-matched control group and subjected study participants to a randomized dietary protocol that adheres to recent recommendations.^18^ However, certain study limitations need to be considered. The small sample size generates the possibility of a type II error. Notwithstanding possible effects on BP, volume and hemodynamic responses in healthy controls that could have been observed with a larger sample size, effect sizes in patients with type 1 diabetes were more pronounced and homogeneous, suggesting a more pronounced salt-sensitive phenotype. It should be noted that this study is not established to provide causal assessment between the observed BP increase and responses in body fluid composition and hemodynamics, among others, since there were no sequential measurements before and during BP responses. For example, it has been shown that increases in plasma volume^33 50^ or CO do not automatically lead to BP increases (reviewed in Kurtz *et al*^14^). Our study serves as a hypothesis-generating study that precisely characterized responses to dietary salt loading in patients with type 1 diabetes. For underlying mechanisms, future studies specifically aimed at this question should be performed. Also, since only male subjects were included, future studies are needed to explore whether our results can be extrapolated to females. Lastly, we did not control fluid intake; therefore, underlying reasons for the observed differences in urine volume cannot be identified with certainty.

In conclusion, we demonstrated that young normotensive normoalbuminuric male patients with type 1 diabetes are more sensitive to the effects of salt intake on BP than healthy individuals. The salt-sensitive BP rise in patients with type 1 diabetes was accompanied by significant increases in plasma volume, CO, HR, and NT-proBNP. Future studies are needed the scrutinize underlying mechanisms—like sodium storage capacity—in order to be able to ultimately unravel the susceptibility to hypertension and cardiovascular risk in patients with diabetes.
