# Supplementary material for: Effect of high-salt diet on blood pressure and body fluid composition in patients with type 1 diabetes: randomized controlled intervention trial
Source: BMJ Open Diabetes Res Care. 2020 May 12;8(1):e001039. doi: 10.1136/bmjdrc-2019-001039 (PMC7228471; doi:10.1136/bmjdrc-2019-001039)
Supplement: Supplementary data [file bmjdrc-2019-001039supp001.pdf]

SUPPLEMENTAL MATERIAL

Supplemental Table 1. Baseline characteristics at screening (screening visits took place before commencement of the dietary interventions). Data are depicted as mean (SD). BMI, body mass index. BP, blood pressure. eGFR, estimated glomerular filtration rate.

	**Type 1 diabetes patients (n=8)**	**Healthy controls (n=12)**	**p-value**
**General characteristics**			
Age (years)	28.1 (5.8)	22.7 (4.1)	<0.05
Length (cm)	184.3 (5.0)	185.6 (6.4)	0.62
Weight (kg)	77.4 (9.5)	75.7 (6.8)	0.66
BMI (kg/m^2)	22.8 (2.5)	22.0 (2.2)	0.50
**Office BP**			
Systolic BP (mmHg)	124 (6)	121 (8)	0.41
Diastolic BP (mmHg)	64 (7)	59 (6)	0.13
Pulse (bpm)	56 (6)	61 (7)	0.12
**Plasma**			
Hb (mmol/L)	9.5 (0.6)	9.3 (0.5)	0.43
Ht (L/L)	0.45 (0.01)	0.43 (0.02)	<0.05
Sodium (mmol/L)	139.0 (1.3)	140.0 (1.8)	0.19
Potassium (mmol/L)	4.4 (0.3)	4.2 (0.2)	0.08
Creatinine (umol/L)	71.5 (9.3)	81.3 (9.5)	<0.05
Urea (mmol/L)	4.8 (1.1)	5.4 (1.1)	0.24
eGFR (ml/min/1.73m^2) (CKD-EPI)	120 (9.57)	115 (12.11)	0.29
Osmolality (mOsm/kg)	295.4 (6.1)	286.6 (16.4)	0.17
Glucose (mmol/L)	8.1 (4.1)	4.7 (0.6)	0.07
HbA1c (mmol/mol)	58.1 (9.7)	-	-
**24h Urine**			
Volume (mL/24h)	2435 (1134)	1624 (588)	<0.05
Osmolality (mOsm/kg)	537 (211)	664 (227)	0.22
Creatinine (mmol/24h)	16 (4)	16 (3)	0.65
Sodium (mmol/24h)	193 (81)	166 (71)	0.45
Potassium (mmol/24h)	74 (39)	71 (16)	0.81
Urea (mmol/24h)	472 (110)	485 (203)	0.91
Albumin (mg/24h)	5.7 (2.8)	5.4 (3.2)	0.79
Duration of diabetes (years)	15 (7)	-	-

Supplemental Figure 1. Individual values of 24 hour urine sodium.

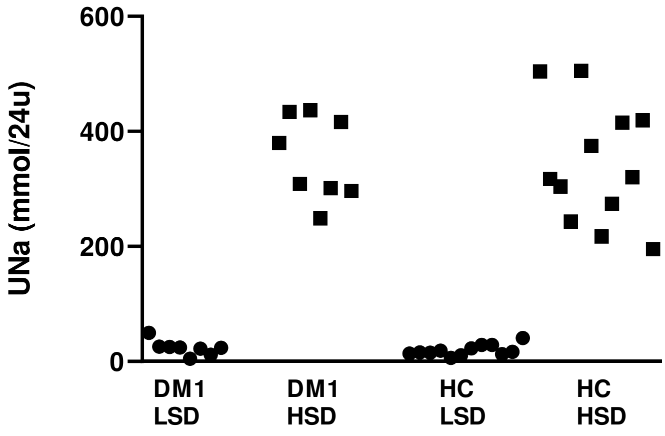

UNa, urinary sodium excretion. DM1, type 1 diabetes patients. HC, healthy controls. LSD, low salt diet. HSD, high salt diet.
